# Population density, bottom-up and top-down control as an interactive triplet to trigger dispersal

**DOI:** 10.1038/s41598-022-09631-w

**Published:** 2022-04-02

**Authors:** Bianca Kreuzinger-Janik, Birgit Gansfort, Christoph Ptatscheck

**Affiliations:** Animal Ecology, Bielefeld University, Konsequenz 45, 33615 Bielefeld, Germany

**Keywords:** Ecology, Ecology

## Abstract

Dispersal reflects the trade-offs between the cost of a change in habitat and the fitness benefits conferred by that change. Many factors trigger the dispersal of animals, but in field studies they are typically not controllable; consequently, they are mostly studied in the laboratory, where their single and interactive effects on dispersal can be investigated. We tested whether three fundamental factors, population density as well as bottom-up and top-down control, influence the emigration of the nematode *Caenorhabditis elegans*. Nematode movement was observed in experiments conducted in two-chamber arenas in which these factors were manipulated. The results showed that both decreasing food availability and increasing population density had a positive influence on nematode dispersal. The presence of the predatory flatworm *Polycelis tenuis* did not consistently affect dispersal but worked as an amplifier when linked with population density with respect to certain food-supply levels. Our study indicates that nematode dispersal on small scales is non-random; rather, the worms’ ability to perceive environmental information leads to a context-dependent decision by individuals to leave or stay in a patch. The further use of nematodes to gain insights into both the triggers that initiate dispersal, and the traits of dispersing individuals will improve the modeling of animal behavior in changing and spatial heterogenous landscapes.

## Introduction

Dispersal is a decisive factor in the temporal and spatial distributions of species and therefore in the diversity and stability of biotic communities. It is described as the movements of an organism that ultimately results in gene flow between or within populations. The three sequential stages of dispersal are *emigration*, *transfer*, and *immigration*^1^. By entering more suitable habitats and avoiding unfavorable ones, dispersing species can increase their fitness. Nonetheless, the move to another habitat has its costs, including the energy invested in physical development (e.g., suitable body size or dispersal structures such as wings) or locomotion rather than in activities such as foraging^2^. Furthermore, during their transfer, dispersing species are at higher risk of predation, starvation, or dehydration if no suitable location is found^2^.

The factors that trigger emigration include internal factors (e.g., morphology, physiology), behavior, and especially environmental factors (^3,4^ and references therein), such as climate, habitat quality, the presence of predators or prey, and competition, all of which may determine the temporal and spatial fluctuations of a population^4–7^.

Numerous studies have identified population density as a fundamental factor triggering emigration, as it determines the degree of inter- and intraspecific competition or inbreeding. Positive density-dependent dispersal, in which increasing population density causes more individuals to leave the habitat, has been demonstrated in invertebrates and vertebrates (e.g.^8–12^ and references therein). However, some studies have reported a negative relationship between density and dispersal (e.g.^9,13^). For those populations, living in high-density groups either offers a fitness advantage (e.g., protection against predation) or its adverse effects are compensated by other factors. Resource availability, such as breeding sites^14^ and especially the amounts of food, have been well investigated in this context. For example, while a low level of suitable food increases the frequency and extent of emigration, sufficient food supplies can have the opposite effect even at higher population densities (e.g.^11,12,15–18^). A sufficient food supply can be a prerequisite for effective dispersal because well-fed, healthy individuals who have completed their physical development will be more likely to emigrate and will better survive the transfer than their malnourished and underdeveloped counterparts^19–21^. Besides population density and the availability of food resources, the presence of a predator can be an important driver of the dispersal of its prey, as described in numerous unicellular organisms, invertebrates, and vertebrates^22–26^. However, the effect can be strongly prey-density-dependent, with the emigration rate being highly pronounced at low densities but reduced at high densities^25^. In the latter study, this density-dependent switch was explained by a change in the individual predation risk, which was high when the prey density was low, thus triggering dispersal to more suitable sites, and low when the density was high, as the dilution effect (the undetectability of the individual in the crowd) reduced the predation risk and outweighed the adverse impact of competition (see also^27^). Dispersal can therefore be regarded as a multifactorial process, one that includes both the factors discussed above but also interspecific interactions, temperature, age structure, sex ratio, hormones, etc.^28,29^. The scientific challenge is to disentangle the relative importance of those factors both individually and in combination.

As the most common metazoans worldwide, nematodes colonize nearly all substrates (e.g., sediments, soil, moss, macrophytes) in freshwater, marine, and terrestrial habitats^30^. They are essential contributors to trophic interactions, nutrient cycles, and energy fluxes. While their small size accounts for their diverse passive dispersal modes, free-living nematodes are also able to spread by locomotion and actively colonize new habitats^31^. Depending on the species, by active crawling nematodes can cross distances of 1–3 cm per minute in/on agar plates (reviewed by Ptatscheck and Gansfort^32^). So far, few studies have shown that the active dispersal of nematodes is condition dependent. Studies of a marine species complex in experimental two-patch systems demonstrated that nematode dispersal behavior responds to inter- and intraspecific competition as well as the food concentration^10,16^. In the model species *Caenorhabditis elegans*, a study measuring the spread of individuals between patches on agar plates showed the positive density-dependence of dispersal. In freshwater sediments, the presence of potential invertebrate predators was shown to induce changes in the vertical distribution of different nematode species^33^ suggesting an active dispersal strategy in response to predatory pressure.

However, these insights are only a first step in understanding the drivers of dispersal. Standardized laboratory experiments investigating the triggers of nematode emigration are necessary to validate the above-described effects and examine their interactive impacts on dispersal. A good candidate for this purpose is the terrestrial nematode species *C. elegans*, one of the best studied model organisms on Earth. *C. elegans* is a 99.9% self-fertilizing hermaphrodite^35^, such that the impact of its mating behavior can mostly be neglected in studies of emigration triggers. Other properties that make *C. elegans* an ideal test organism in laboratory experiments are its easy cultivation and handling and the ready availability of large numbers of individuals. The physical prerequisites for condition-dependent dispersal, i.e., a chemosensory ability have been described in detail in *C. elegans*^34^ and chemical cues associated with food, danger, or other individuals have been identified as essential drivers of the worm’s movement behavior.

Thus, in this study *C. elegans* served as the model organism to investigate the effects of organismal density, food availability, and the presence of a predator, both individually and in combination, as triggers of emigration. Our experiments were conducted in two-patch systems, similar to the method employed by Fronhofer et al.^26^ in unicellular organisms and metazoans, such as crustaceans, mollusks, arthopods, and vertebrates, and successfully adapted to nematodes by Meester et al.^10,16^.

We hypothesized that nematode emigration increases in response to a lower availability of food resources (H1), to the presence of a predator (H2), and to a higher population density (H3). We also examined whether these three effects work additively or interactively. Specifically, as population density is a well-established fundamental factor driving nematode dispersal, we further predicted that (H4) the influence of food availability and predation on dispersal is mitigated by an increasing nematode density.

## Material and methods

### Nematode culture

*Caenorhabditis elegans* (var. Bristol, strain N2) was cultivated on agar plates overgrown by *Escherichia coli* (OP50). An agar solution made up of 488 ml of water, 8.5 g agar, 1.25 g peptone, 1.5 g NaCl_2_ (autoclaved at 121 °C for 10 min), 0.5 ml 1 M CaCl_2_, 0.5 ml 1 M MgSO_4_, 12.5 ml 1 M K-PO_4_ (pH 6.0) and 0.5 ml cholesterol-sol [5 mg/ml ETOH] was prepared and poured into petri dishes. After the solution had hardened, 300 µl of LB-medium (10 g peptone, 5 g yeast extract, 10 g NaCl_2_ in 1 l of deionized water) containing *E. coli* (grown for 24 h at 37 °C) was distributed on the surface and the plates were incubated for 24 h at 37 °C. After a nematode-containing piece of agar had been added to each petri dish, the nematodes were cultivated for 7 days at 20 °C in a heat cabinet and then harvested by washing the plates with K-medium (3.1 g NaCl_2_, 2.4 g KCl in 1 l of deionized water). Individuals retained on a 35-µm sieve (large nematodes) and those that passed through a 5 µm sieve (small nematodes) were used in the experiments. For both size classes, three different densities were prepared (500, 1000, and 2500 nematodes per 1.5 ml of K-medium).

### Testing arenas

**Figure 1:**
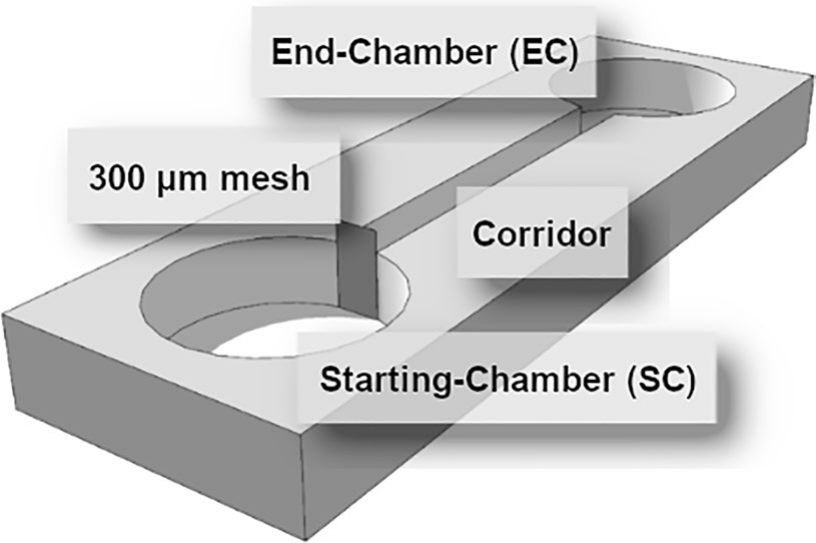
Design of the testing arenas.

All experiments were performed in testing arenas consisting of two round chambers (Ø 5 cm) made from 1.3-cm plexiglass and connected by a 10-cm-long corridor (1 cm in width) that had been milled to a depth of 1 cm (Fig. 1). These dimensions were determined in previous nematode migration studies^10,16,36^ and are based on the actual velocity (crawling or swimming) of different nematode species. For example, *C. elegans* is able to move up to 15 mm per minute^36^. One chamber served as the starting-chamber (SC), where the nematodes were added at the beginning of the experiment, and the other as the end-chamber (EC) into which the emigrating nematodes arrived.

**Figure 2:**
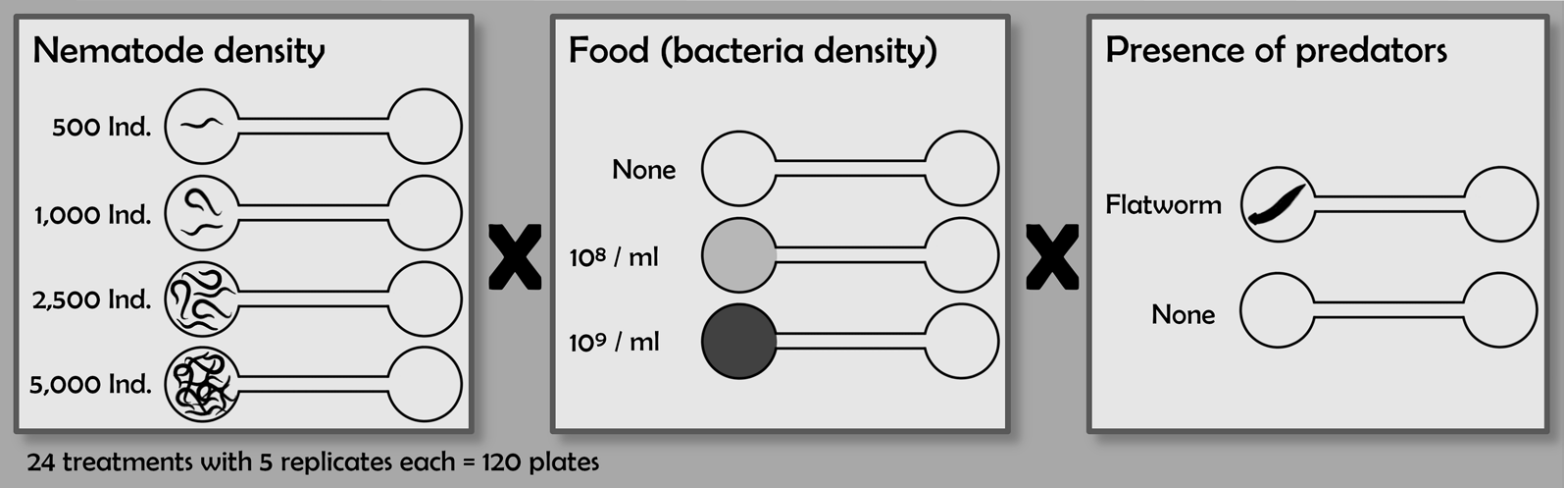
Experimental design to test the effects of the combined factors of nematode density (500, 1000, 2500 and 5000 nematodes per SC), food availability (no bacteria, 10^8^ and 10^9^ cells ml^−1^), and the presence or absence of a predator in the SC.

Before the start of the experiments, the SCs were separated by gluing small pieces (1.5 cm × 1.5 cm) of 300-µm polyamide fabric in front of the corridors with a droplet of agar (Fig. 1). Previous experiments showed that the mesh did not restrict nematode movement compared to treatments without mesh (Supplementary Information, Fig. S1). The medium pipetted into the corridor and EC consisted of 9.2 ml NGG (nematode growth gelrite, 1.26 g gelrite in 500 ml of deionized water), 2.3 ml of K-medium and 2.3 ml of salt-solution (0.44 g CaCl2, 0.74 g MgSO4 in 500 ml of deionized water) prepared in a centrifuge tube and vortexed for a few seconds. Due to its high viscosity, the medium was unable to flow through the mesh to form a 3-dimensional matrix. The SCs of the arenas were prepared according to the investigated treatments, in which the effects of different nematode and bacterial densities and the presence or absence of a predator were tested (Fig. 2). Five replicates per treatment were prepared.

### Nematode density

After the EC and corridor had been prepared, the SC was filled with NGG containing a defined number of nematodes as follows: Six ml NGG, 1.5 ml salt-solution, and 1.5 ml K-medium containing different densities (500, 1000, 2500, or 5000) of small or large *C. elegans* (see “Nematode culture”) were mixed in centrifuge tubes and pipetted into the chamber. This resulted in the homogeneous distribution of the nematodes within the gelrite. Prior to the start of the experiment, we prepared a stock solution containing the nematodes and counted the number of nematodes in 20 10-µl drops in order to calculate the amount of suspension needed for each treatment before adding K-Medium to obtain the 1.5-ml needed for the experiment. To avoid variations in the number of nematodes in the SCs, five replicates were prepared for each density prior to the start of the experiment, with a maximum deviation of 3.3%.

### Availability of food resources

*E. coli* OP50 was used as the food resource and was cultivated in LB-medium as described above. After 1 day of incubation, the *E.coli* cells were washed with K-medium and resuspended to obtain densities of 10^8^ and 10^9^ cells ml^−1^ (referred to below as low and high bacterial densities), confirmed spectrophotometrically according to the protocol of Muschiol and Traunspurger^37^. The K-medium was then removed by centrifugation and the bacterial pellet resuspended in semifluid NGG^37^ devoid of peptone to prevent additional bacterial growth. The NGG containing bacterial cells were added to the SCs in experiments testing different numbers of nematodes and three concentrations of *E. coli* (none, 10^8^ cells ml^−1^, 10^9^ cells ml^−1^).

### Presence of predators

The predator used in our experiments was the flatworm *Polycelis tenuis*. This species, like other flatworms, exerts strong top-down pressure on both aquatic and terrestrial nematodes (e.g., *C. elegans*)^38–40^. A single *P. tenuis* flatworm was placed in the middle of the SC, on top of the gelrite.

The installed piece of 300-µm polyamide fabric (see “Testing arenas”) prevented the movement of the predators into the corridor but enabled nematode emigration. Controls without predator but with fabric were also prepared to investigate the possible impact of the flatworm on the emigration of *C. elegans* (Supplementary Information, Fig. S1).

### Experimental procedure and nematode counting

Prior to the experiment, we tested whether the proportion of nematodes in the ECs after 6 h differed when large (> 35 µm) or small (< 5 µm) nematodes were supplied to the SCs. The nematode density was held constant at 2500 individuals and no bacterial cells as food were added.

As it was shown that only large nematodes emigrate (see “Results” section), the experiment was run with large individuals for 12 h after the SCs had been prepared. In preliminary experiments, the test chambers were checked once an hour during the first 6 h of the experiment and then after 12 and 24 h. The continuous increase in the number of individuals in the ECs suggested that the movement of nematodes back and forth between the SC and EC was neglectable within the chosen time span of 12 h. Subsequently, the nematodes in the ECs were counted under a stereomicroscope at 40 × magnification.

### Statistical analysis

To test whether large (individuals retained on a 35-µm sieve) or small (individuals passing through a 5-µm sieve) nematodes significantly differed in their tendencies to emigrate, we calculated the percentages of large vs. small nematodes in the EC after 6 h. As the values were not normally distributed (Shapiro-test), a Mann–Whitney *U*-test was performed.

A generalized linear model (GLM) was used to test whether the factors nematode density, food availability (bacterial density), the presence of a flatworm as predator, and interactions thereof significantly influenced emigration strength, measured as the rate at which nematodes reached the EC after 12 h. These dispersal rates were analyzed as ratios of counts in the GLMs with a binomial error structure.

The factors of nematode density (500–5000 individuals) and food availability (none, 10^8^ cells ml^−1^, 10^9^ cells ml^−1^) were treated as continuous variables; the factor predator was treated as a categorical variable. Since the ratio of the sample size and number of estimated parameters included in the model was < 40, a second-order Akaike information criterion (AICc) was used to evaluate the model (as recommended by Burnham and Anderson^41^). In addition, a forward selection procedure using likelihood ratio tests for nested models was performed to determine the significances of the individual predictors in the model, using χ^2^ tests^42^. Only interactions whose respective main effects were entered into the model were included. All statistical analyses were performed in the *R* environment^43^, using the package ‘AICcmodavg’^44^.

## Results

### Large vs. small nematodes

A possible difference between the emigration strength of small vs. large nematodes was tested under a constant nematode density of 2500 individuals and without the addition of bacterial cells as food. The results showed that after 6 h there was almost no emigration of small nematodes from the SCs (0.05 ± 0.07%) whereas 5.73 ± 1.60% of the initially supplied large nematodes arrived in the ECs. The difference was significant (W = 16, n = 4, p = 0.029, Mann–Whitney *U*-test). Based on this result, only large nematodes were used in further experiments.

### Effect of nematode density, food availability and predator

The GLM that best fitted nematode dispersal rates is summarized in Table 1 (for model selection, see Table S1). This model best reflected the experimental outcome, as the ∆AICc of the second best model was 48.4^41^ (Table S1). This GLM was the full model, thus including the three main effects and both all two- and the three-way interactions.

**Table 1:** A generalized linear model using the binomial family.

Coefficients	Estimate	Std. error	t value	p
Intercept	− 3.0370	0.0369	− 82.43	**< 0.001**
Bacteria	− 0.8680	0.0425	− 20.40	**< 0.001**
Density	0.0005	< 0.0001	52.88	**< 0.001**
Flatworm	− 0.5473	0.0559	− 9.79	**< 0.001**
Bacteria: density	< 0.0001	< 0.0001	− 1.95	0.0514
Bacteria: flatworm	− 0.0340	0.0626	− 0.54	0.5873
Density: flatworm	0.0002	< 0.0001	12.98	**< 0.001**
Bacteria: density: flatworm	0.0001	< 0.0001	7.12	**< 0.001**
Null deviance: 36,808.7 on 119 df; Residual deviance: 7195.1 on 112 df				

Significant values are in bold.

The response variable was the proportion of nematode individuals in the ECs after 12 h; the predictors were: nematode density [continuous: 500, 1000, 2500, 5000], bacterial density in the SCs [continuous: none, 10^8^ cells ml^−1^, 10^9^ cells ml^−1^] and the absence/presence of a flatworm [2 factors]. The presented model is the one that best fit the data during model selection (details in Table S1). The estimated intercept was based on the absence of both food and predator and under a nematode density of 500.

**Figure 3:**
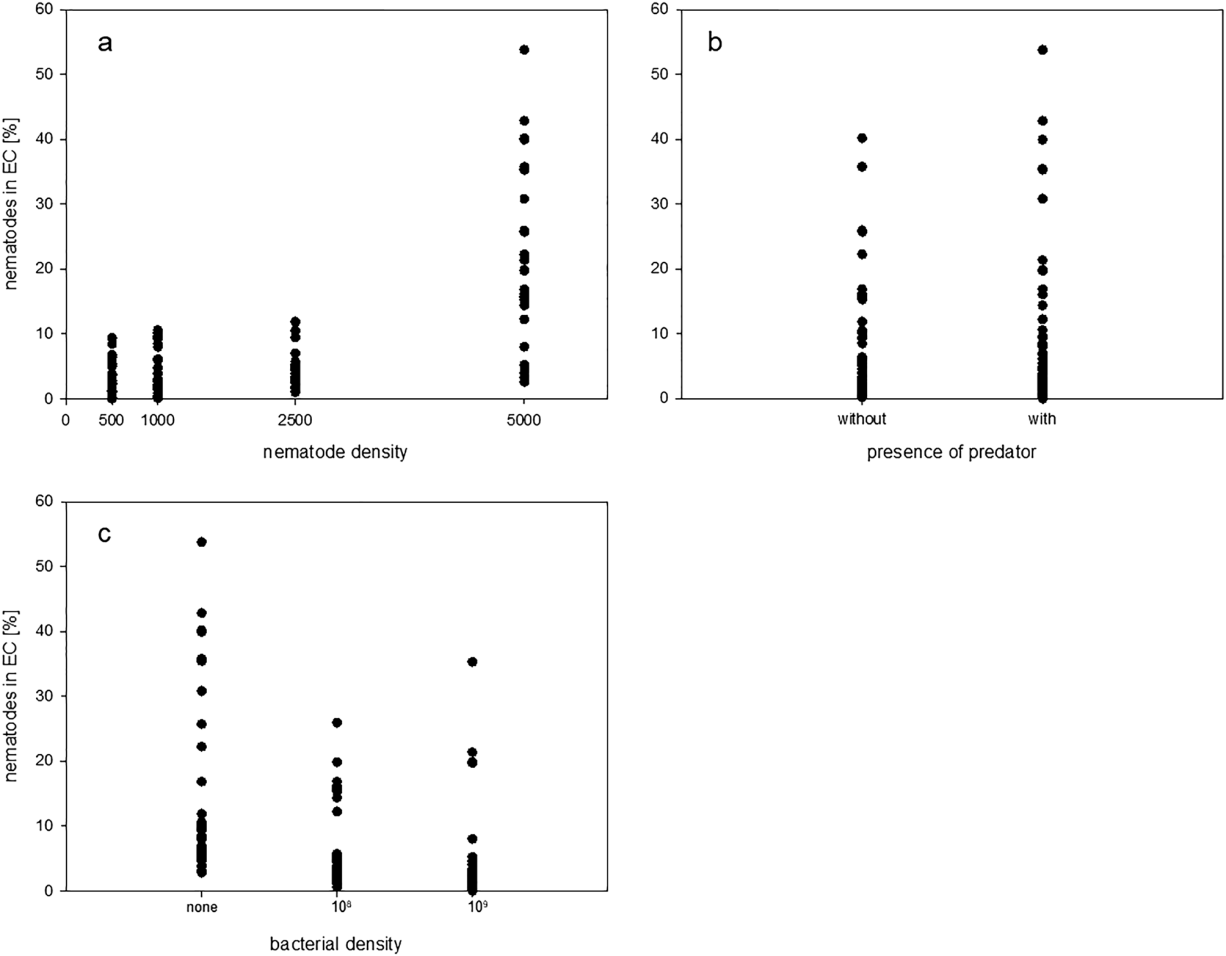
Percentages of all nematodes in the SCs that reached the ECs after 12 h for the single predictors (**a**) nematode density, (**b**) presence of predator, and (**c**) bacterial density.

The bacterial density in the SCs significantly affected the emigration intensity of *C. elegans* (Table 1). With increasing bacterial density, the percentage of nematodes arriving in the ECs decreased, from 13.98 ± 13.21% in the absence of bacteria to 6.48 ± 6.42% and 4.15 ± 7.22% in the presence of 10^8^ *E. coli* cells ml^−1^ and 10^9^ *E. coli* cells ml^−1^, respectively (Fig. 3c).

The density of *C. elegans* in the SCs at the start of experiment was significantly and positively related to the nematodes dispersal rate (Table 1). However, the slope was relatively flat, as the crucial increase in the dispersal rate occurred at the highest density of 5000 (21.2 ± 13.1), while at lower densities emigration strength did not differ remarkably (500: 3.2 ± 2.5%; 1000: 4.2 ± 3.5%; 2500: 4.3 ± 3.1; Fig. 3a).

**Figure 4:**
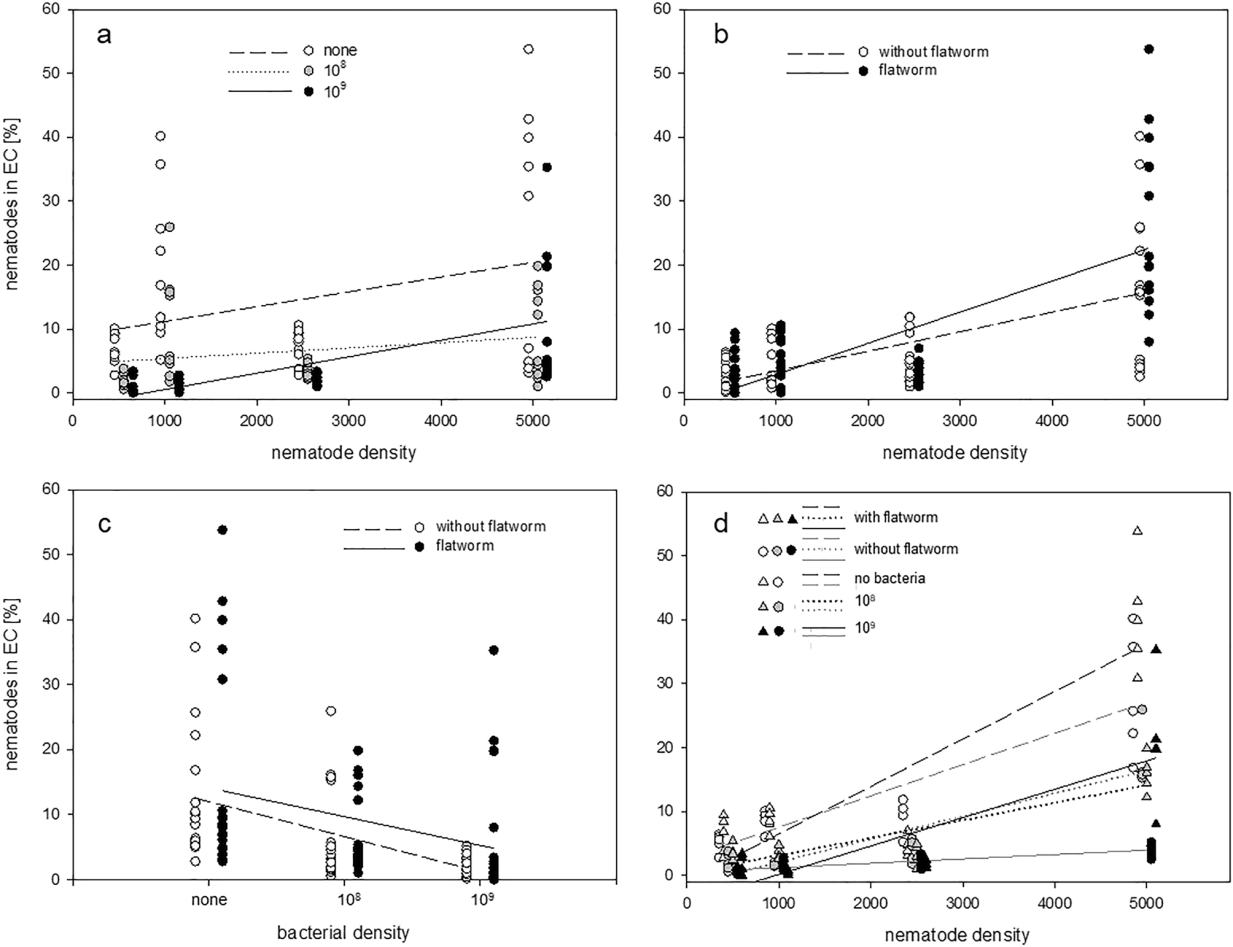
Percentages of all nematodes in the SCs that reached the ECs after 12 h for the combined predictors (**a**) nematode density and bacterial concentration, (**b**) nematode density and presence of predator, (**c**) bacterial density and presence of predator, and (**d**) the triple interaction of all predictors.

Further, the significant interactions between nematode density and bacterial density (Table 1, Fig. 4a) indicated that the strength of the increase in the dispersal rate with increasing nematode density depended on the level of food availability.

According to the model, the presence of a predator slightly reduced the number of nematodes reaching the EC after 12 h (Table 1). This might be counterintuitive, given that the overall mean dispersal rate was higher when a flatworm was present (no flatworm: 7.15 ± 8.46%; with flatworm: 9.26 ± 11.79%, Fig. 3b). However, the flatworm’s effect widely varied with respect to the different food and especially to the different density conditions (Table S2). Specifically, under the lowest and highest densities the flatworm had a positive effect on dispersal but under middle densities its effect was negative (Table S2, Fig. 4b) although under a low bacterial density these relationships were again reversed (Table S2, Fig. 4c,d). These varying effects were manifested in the significance in the model of both the two-way interaction between the flatworm’s presence and density and the three-way interaction between the latter factors and food availability (Table 1).

## Discussion

Our experiments demonstrate that the active dispersal of *C. elegans* is not random but instead reflects a trade-off between the costs of transfer and the expected fitness benefits, as observed in the SCs vs. the ECs^2,45^. Specifically, our results showed a clear negative effect of food availability and a clear positive effect of very high population densities on the emigration strength of *C. elegans*. Furthermore, the generally high degree of interaction between the three single triggers (food, density, and predation) suggests that predictions of organismal dispersal based on single factors will be prone to errors and may call into question the validity of extrapolations from and to field metacommunities.

### Bottom-up effects

In line with our initial hypothesis (H1), the increase in bacterial availability had a constant and strong inhibitory effect on the emigration frequency of *C. elegans*, such that dispersal rates were almost four times lower in treatments with high food availability than in those in which food bacteria were absent. In the context of fitness maximization, our findings suggest that a high bacterial density implies a high habitat quality and thus no need for the nematodes to leave the patch, as target patches of higher quality cannot be expected. When supplied with an unlimited amount of high-quality food, nematodes eventually become satiated, stop eating and moving, and become quiescent^46^. Indeed, a reduction in the undulatory movements of worms in the SCs containing bacteria was clearly recognizable, especially in the 10^9^ cells ml^−1^ treatment. Similar reductions were not visible in the corridors and ECs. These observations are consistent with the behavioral strategies of *C. elegans* reported by Shtonda and Avery^47^. In that study, the authors quantified the effects of food quality on the locomotion of *C. elegans* and showed that nematode movement in the presence of high-quality food is characterized by many interruptions, while under lower food quality the movement of the worms is more directed, faster, and more persistent.

A sharp increase in dispersal with food limitation is seen in a wide range of animals, from protists, to earthworms, crustaceans, and insects, and to vertebrates including lizards, fish, and birds^12,26,48,49^. However, other studies in which either other *C. elegans* breeding lines obtained from different locations or cryptic species were used revealed deviations in nematode emigration behavior in response to food resources^16,50^. The authors of those studies concluded that nematode dispersal is complex and not describable by a single factor. By contrast, the two-way interactions including bacterial density examined in our study were not significant, indicating that food availability is a main trigger of nematode dispersal and that it has a consistent and predictable impact even under varying population densities and predatory pressures (Fig. 4a,c).

### Top-down effects

In contrast to the consistency of the changes in the dispersal rates of *C. elegans* in response to bacterial availability, the presence of the predator *P. tenuis* had varying, highly condition-dependent effects. Therefore, our hypothesis (H2) was not supported by the experimental outcome, as higher dispersal rates were generally not observed in treatments that included a flatworm. Rather, the dispersal behavior of the nematode under predatory pressure was also shown to depend on the level of intraspecific competition and the food supply, as indicated by the significant two- and three-way interactions. Thus, the increase in the dispersal rate in response to a higher population density was steeper when a flatworm was present (Fig. 4b), although this increase in the slope was less pronounced when bacterial food was withheld or provided at only a low level (Fig. 4d).

Those highly interactive effects indicate that the presence of *P. tenuis* alone does not result in a strong fitness disadvantage for *C. elegans* that would outweigh the costs of dispersal. A previous study determined that *P. tenuis* is able to consume 50 adult *C. elegans* individuals within 3 h, a rate high enough to enhance the risk for individual nematodes to be caught^40^. However, after 12 h we found no reduction in the number of nematodes by the flatworm. This result can be explained by the distribution of the organisms in/on the NGG; thus, while the nematodes were dispersed throughout the gelrite, the flatworm mainly remained on the surface, which hampered its effective ingestion of nematodes. Further, flatworm mucus has the potential to enhance bacterial growth^51^ such that the negative effect of *P. tenuis* on *C. elegans* may have been weakened by an unintentionally positive, attractive effect of the flatworm.

A study investigating the role of predatory pressure on the dispersal of very different animal groups found no evidence of a general negative or positive top-down effect on dispersal. Rather, dispersal was shown to depend on the spatial use of both predator and prey and on the dispersal habitat (aerial, aquatic, or terrestrial)^26^. As noted by the authors of that study, “species that routinely use more space than their predators showed increasing dispersal in response to predation, especially in aquatic environments”^26^. On the one hand, the home range of *C. elegans* is unlikely to be larger than that of the over ten times larger flatworm, but on the other hand, the movement of the predator was reduced by the semifluid medium used during the experiment.

However, in our study, under high intraspecific competition (i.e., high population density), the predator acted as an amplifier of dispersal, an effect that was strongest under the highest food-supply level. Baines et al.^24^ examined dispersal as a function of body condition and predation and hypothesized that high-condition individuals have the greatest ability to respond to a predation risk. In support of their hypothesis, our study showed that the increased emigration rate of *C. elegans* in response to density was steepest in the presence of a predation risk and under a very high bacterial concentration (Fig. 4b,d). Moreover, the potential of flatworm mucus to enhance bacterial growth and thereby attract nematodes^51^ may become irrelevant when a high bacterial density (in our study, 10^9^ cells ml^−1^) is offered.

### Density dependent emigration

The effect of density on dispersal may be positive or negative. In the former, competition induces individuals to emigrate while in the latter social crowding (e.g., protection in a large group) hampers emigration^9^. In our study, the emigration of *C. elegans* showed a significantly positive density dependence, in line our initial hypothesis (H3). However, the increase was characterized by a flat slope, which implied that it was determined especially by a very high nematode density (n = 5000), under which the average dispersal rate quintupled (Fig. 3a).

This result was consistent with the fact that *C. elegans* is predominantly self-fertilizing^35^ and the N2 strain used in our experiments is solitary (no aggregation in culture plates; compare with Gloria-Soria and Azevedo^52^). Positive density-dependent dispersal is common and has been demonstrated in a broad range of animal groups^11^, including birds, mammals^9^ and insects^53,54^. Other studies conducted with nematodes showed that the propensity to disperse increases with increasing population density (marine species^10^; solitary and gregarious strains of *C. elegans*^52^). Meester et al.^10^ proposed that nematodes perceive a “density threshold” above which individuals start to disperse, as also reflected by our data. According to the theoretical model developed by Travis et al.^8^, density-dependent emigration implies relatively high dispersal costs for the studied species such that individuals tend to remain in a patch until competition exceeds a threshold that is above the equilibrium of possible subpopulations. The costs of dispersal include both the risk of mortality while moving and not finding a suitable target patch^8^. For nematodes, these risks are high due to the small distance that nematodes can actively reach^31^. Nonetheless, *C. elegans* is able to reduce both risks through its ability to sense the quality of a nearby patch^55,56^ and to produce dauer larvae that facilitate long-distance dispersal^50^. Due to our experimental design, with no food in the ECs, the motivation for nematodes to leave the SCs may have been low while the duration of the experiment was too short to allow the production of dauer larvae. Our study therefore measured the dispersal of adult non-dauer larvae, a condition that has not been well-studied^50^. In natural soil habitats, nematode densities should be lower than in the experimental set-up used in this study (e.g., Freckman et al.^57^ found maximum densities of 160 individuals/20 cm^3^). However, comparisons are difficult because nematode densities in soil are usually reported per sampled area regardless of the sampled depth. In laboratory cultures, *C. elegans* densities are usually substantially higher (up to 35,000 ind. on 20 cm^2^, own counts). Together, these factors could explain the relatively small influence of population density in our experiments and the lack of evidence supporting (H4), that the influence on dispersal of food availability and predation is mitigated by intraspecific competition.

## Conclusion

There is increasing evidence that dispersal is a non-random, highly complex, and multicausal process^58^ in which local and regional dynamics are linked. Our experiments support this observation, as they showed that dispersal of the model organism *C. elegans* is affected not only by single factors, such as resource availability and population density, but also by a combination of factors acting synergistically. Thus, predictions about dispersal based on single-factor experiments will not be applicable to field conditions. However, there is little empirical evidence for multi- causal interactions because multifactorial tests of the interactive role of dispersal factors are lacking. This can be explained by the technical challenges associated with the need to control multiple factors under natural conditions. Also, in our full-factorial experimental design, data interpretation was impeded: (1) by the generally large variations in nematode active dispersal rates, as even under null conditions (without food and predator) the rates varied by twofold among the five replicates of a nematode density of 5000; and (2) by the high degree of interaction between the three investigated factors, such that a single factor’s role could not be distinguished. We therefore recommend the use of high replicate numbers in future studies of nematode dispersal triggers.

Although nematodes are passive dispersers over larger distances^31^ our results show that context-dependent active dispersal occurs also on small scales. Therefore, nematodes, with their small size and short generation times, are a valuable organism in laboratory studies of dispersal. Moreover, the differential response of nematode dispersal behavior to different environmental conditions^16^ offers a further opportunity to test the effect of different traits at the species and individual levels (i.e. dispersal syndromes) and thereby gain broader insights into dispersal processes. The results of such studies can be used to model animal behavior in response to the environmental changes arising from increasing habitat fragmentation. This knowledge is relevant in both ecological forecasting and conservation management.

## Supplementary Information

Supplementary Figure 1.

Supplementary Tables.

## Data Availability

The datasets generated during and/or analysed during the current study are available from the corresponding author on reasonable request.
