# Supplementary material for: Population density, bottom-up and top-down control as an interactive triplet to trigger dispersal
Source: Sci Rep. 2022 Apr 2;12:5578. doi: 10.1038/s41598-022-09631-w (PMC8976845; doi:10.1038/s41598-022-09631-w)
Supplement: Supplementary file 1 — Supplementary Figure 1. [file 41598_2022_9631_MOESM1_ESM.pdf]

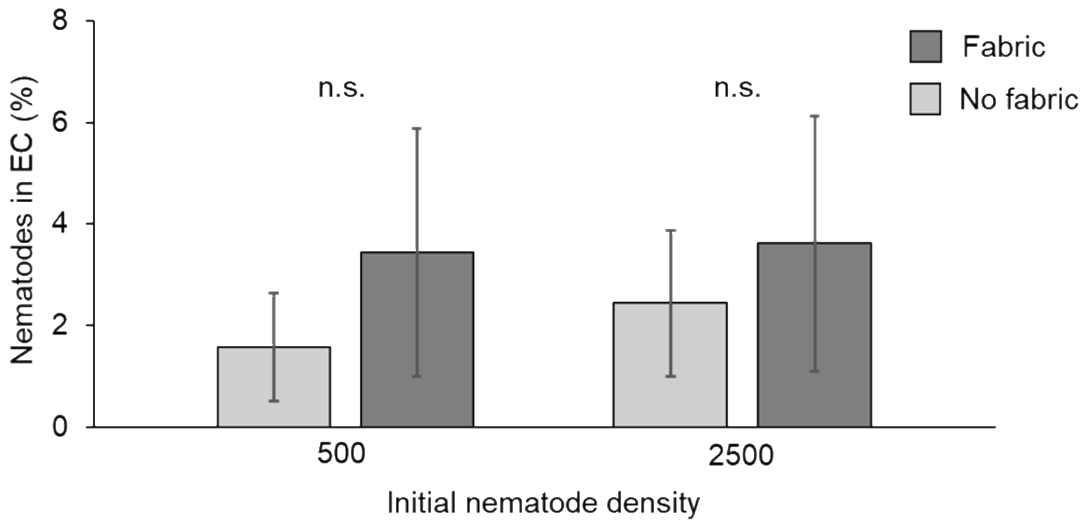

Figure S1: Mean percentages of all nematodes found in the testing arenas which reached the ECs after 6 hours with a fabric of a 300µm-mesh size (dark gray) or no fabric (light gray) separating the SCs from the corridor (see Figure 1) with regard to an initial nematode density of 500 and 2500 individuals respectively. The differences between dispersal rates were tested with a t-test (after checking normality of data), n.s.: p>0.05
