# Supplementary material for: Population density, bottom-up and top-down control as an interactive triplet to trigger dispersal
Source: Sci Rep. 2022 Apr 2;12:5578. doi: 10.1038/s41598-022-09631-w (PMC8976845; doi:10.1038/s41598-022-09631-w)
Supplement: Supplementary file 2 — Supplementary Tables. [file 41598_2022_9631_MOESM2_ESM.docx]

**Table S1:** Model selection results for generalized linear mixed models using the binomial family. The response variable was the dispersal rate, the possible predictors were nematode density (continuous: 500-5000); bacterial density [continuous: 0 (no bacteria) - 1 (10^8^) - 2 (10^9^) cells ml^−1^] and presence of a flatworm (2 factors: without and with flatworm). In the likelihood ratio test, the “best” model according to AICc (in the first line of each section, highlighted in bold) was tested against each possible model including one additional predictor (forward selection).

| **Formula** | **Df** | **Deviance** | **AIC** | **LRT** | **Pr(>Chi)** | **AICc** | **DeltaAICc** |
| --- | --- | --- | --- | --- | --- | --- | --- |
| **1** |  | 36809 | 37522 |  |  |  |  |
| B | 1 | 28277 | 28992 | 8532.2 | <0.001 | 28991.63 | 21068.207 |
| D | 1 | 18906 | 19621 | 17903 | <0.001 | 19620.96 | 11697.537 |
| R | 1 | 35600 | 36315 | 1208.9 | <0.001 | 36314.84 | 28391.417 |
| **D** |  | 18905.8 | 19621 |  |  |  |  |
| D+B | 1 | 9841.8 | 10559 | 9064.1 | <0.001 | 10559.01 | 2635.587 |
| D+R | 1 | 17623 | 18340 | 1282.7 | <0.001 | 18340.37 | 10416.947 |
| D+B | 1 | 9841.8 | 10559 |  |  |  |  |
| D+B+R | 1 | 8500.4 | 9219.4 | 1341.4 | <0.001 | 9219.761 | 1296.338 |
| D+B+D:B | 1 | 9790.4 | 10509 | 51.371 | <0.001 | 10509.78 | 2586.357 |
| **D+B+R** | 1 | 8500.4 | 9219.4 |  |  |  |  |
| D+B+R+D:B | 1 | 8451.4 | 9172.4 | 49.02 | <0.001 | 9172.915 | 1249.492 |
| D+B+R+D:R | 1 | 7780.4 | 8501.4 | 720.04 | <0.001 | 8630.227 | 706.804 |
| D+B+R+R:B | 1 | 7908.7 | 8629.7 | 591.71 | <0.001 | 8501.9 | 578.477 |
| **D+B+R+R:B** | 1 | 7908.7 | 8629.7 |  |  |  |  |
| D+B+R+R:B+D:B | 1 | 7723.9 | 8446.9 | 56.455 | <0.001 | 8447.662 | 524.239 |
| D+B+R+R:B+D:R | 1 | 7265.8 | 7988.8 | 514.57 | <0.001 | 7989.545 | 66.122 |
| **D+B+R+R:B+D:R** | 1 | 7265.8 | 7988.8 |  |  |  |  |
| D+B+R+R:B+D:R+D:B | 1 | 7245.8 | 7970.8 | 20.007 | <0.001 | 7971.795 | 48.372 |
| **D+B+R+R:B+D:R+D:B** | 1 | 7245.8 | 7970.8 |  |  |  |  |
| D+B+R+R:B+D:R+D:B+D:B:R | 1 | 7195.1 | 7922.1 | 50.669 | <0.001 | 7923.423 | 0 |

**Table S2**: Differences in means of treatments with and without predator, a negative sign indicates that the dispersal was lower when a flatworm was present and vice versa.

|  | **Nematode density** | | | |
| --- | --- | --- | --- | --- |
| **Bacterial density** | **500** | **1000** | **2500** | **5000** |
| **no** | 1.08 | -0.08 | -5.04 | 12.408 |
| **low** | 1.28 | 2.08 | -0.84 | -1.864 |
| **high** | 1.16 | -1.28 | -0.552 | 16.9 |
